# Reversible alterations in cardiac morphology and functions in a patient with Cushing's syndrome

**DOI:** 10.1530/EDM-14-0038

**Published:** 2014-06-01

**Authors:** Hiroaki Iwasaki

**Affiliations:** 1 Division of Endocrinology and Metabolism, Department of Internal MedicineToshiba Rinkan Hospital7-9-1 Kami-tsuruma, Minami-ku, Sagamihara, Kanagawa, 252-0385Japan

## Abstract

A 45-year-old female was referred for endocrine evaluation of an incidental mass (31×24 mm in diameter) on the right adrenal gland. The patient was normotensive and nondiabetic, and had no history of generalised obesity (body weight, 46 kg at 20 years of age and 51.2 kg on admission); however, her waist-to-hip ratio was 0.97. Elevated urinary free cortisol levels (112–118 μg/day) and other findings indicated adrenocorticotrophic hormone-independent Cushing's syndrome due to right adrenocortical adenoma. Echocardiography before adrenalectomy revealed concentric left ventricular (LV) hypertrophy with a particular increase in interventricular septum thickness leading to impaired systolic and diastolic functions. Upon surgical remission of hypercortisolism, the asymmetric hypertrophy disappeared and the cardiac dysfunctions were considerably ameliorated. Although the mechanism(s) by which excessive cortisol contributes to LV wall thickness remain(s) unclear, serial echocardiography and cardiac multidetector-row computed tomography may support the notion that abnormal fat deposition in the myocardium owing to hypercortisolism appears to be an important factor for the reversible change in the cardiac morphology.

**Learning points:**

- Patients with Cushing's syndrome occasionally exhibit severe LV hypertrophy related to systolic and diastolic dysfunctions although they have neither hypertension nor diabetes mellitus.
- Biological remission of hypercortisolism can normalise structural and functional cardiac parameters and help in differentiating the cardiac alterations induced by excessive cortisol from those induced by other diseases.
- Excessive lipid accumulation within the heart before myocardial fibrosis may be implicated in reversible alterations in the cardiac morphology by Cushing's syndrome.
- Early diagnosis and treatment of Cushing's syndrome appear to be pivotal in preventing irreversible cardiac dysfunctions subsequent to cardiovascular events and heart failure.

## Background

Cushing's syndrome, resulting from excessive autonomous cortisol secretion by the adrenal gland, ectopic adrenocorticotrophic hormone (ACTH) or corticotrophin-releasing hormone secretion by a pituitary or nonpituitary tumour and chronic glucocorticoid therapy, leads to a constellation of complications, including hypertension, diabetes mellitus, cardiovascular disease, stroke and thromboembolism (1). Clinical management of Cushing's syndrome should be carried out particularly with care in identifying cardiovascular risks, and echocardiography and Doppler ultrasonography of the epiaortic vessels are therefore recommended for all patients with Cushing's syndrome at the point of diagnosis (1).

Left ventricular (LV) concentric remodelling with an increased interventricular septum thickness, termed asymmetric septal hypertrophy, is typically a finding of hypertrophic cardiomyopathy due to mutations in the genes encoding sarcomeric proteins; however, it may appear to be a sign of other cardiac and systemic diseases (2). Cushing's syndrome is known to cause various cardiac alterations, including asymmetric septal hypertrophy (3) (4) (5). The changes in the cardiac structure in Cushing's syndrome are associated with the length of disease but not the degree of cortisol excess (6) and occur more often than essential hypertension and other secondary hypertensions such as renovascular hypertension and primary aldosteronism (3).

This report describes a patient with active Cushing's syndrome due to adrenocortical adenoma accompanied by unexplained cardiomegaly over the past 3 years. The patient was not diagnosed with hypertension or diabetes but displayed severe LV concentric hypertrophy with asymmetric septal thickness concomitant to systolic and diastolic dysfunctions. Serial echocardiography revealed that surgical remission of hypercortisolism led to normalisation of parameters for the LV structure along with the recovery of cardiac functions, suggesting the pivotal role of excessive cortisol in the cardiac alterations.

## Case presentation

A 45-year-old female was referred for evaluation of a right adrenal incidental mass on abdominal computed tomography (CT) that was ordered to assess intermittent abdominal discomfort. Three years before the first visit to our department, the patient underwent health check-up at a hospital including electrocardiogram and chest X-ray examination. LV hypertrophy and cardiac enlargement were noted; she was immediately referred to the cardiology department of the same hospital. A cardiologist performed echocardiography and confirmed LV concentric remodelling without specifying its aetiology and recommended regular electrocardiogram, echocardiography and chest X-ray examinations. The patient underwent these examinations every year but took no further medical treatment before adrenalectomy. She also noticed a menstrual disorder (oligomenorrhoea) 2 years before admission; however, emotional liability, sleep disorders and depression were not observed.

The patient had no history of generalised obesity and her body weight on admission was 51.2 kg (BMI, 23.3), although she gradually gained weight by 4 kg for the past 5 years and her waist-to-hip ratio was 0.97 (waist circumference, 78.8 cm) at the time of diagnosis. During admission, her blood pressure was measured several times during the day (in the morning, mid-afternoon and before sleeping) and was found to be between 112/64 and 128/78 mmHg without antihypertensive drugs and salt restriction. Physical examination revealed bilateral malar erythaema, bruising and thin skin. The patient showed no other features, such as apparent accumulation of fat in the face (a moon face) or around the neck (a buffalo hump), hirsutism, acne, prominent purple striae on the abdomen and proximal muscle weakness. Neither crackles nor murmurs were heard from the chest, although Valsalva manoeuvre was positive. Other physical findings were unremarkable.

**Figure 1:**
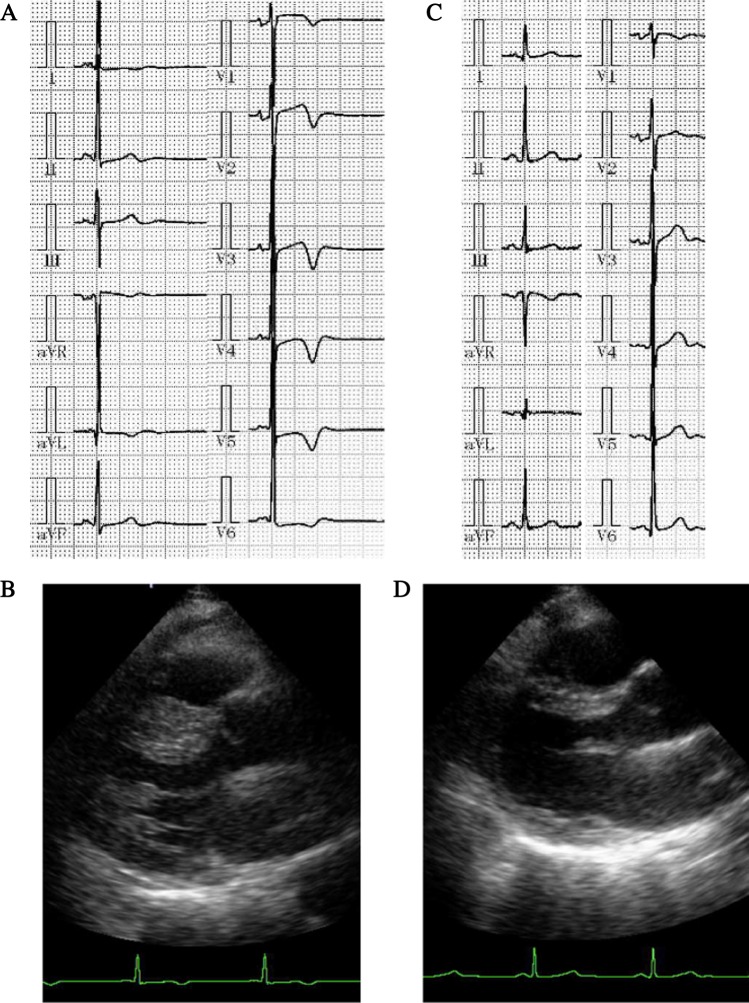
Electrocardiogram and echocardiography before and after adrenalectomy. Electrocardiogram and echocardiography on admission revealed high-voltage QRS complexes, impaired ST segments and T-wave inversion in the broad leads (A) and severe left ventricular concentric hypertrophy (B), all of which were almost within the normal ranges at 8 months after adrenalectomy (C and D).

On admission, chest X-ray revealed cardiac enlargement defined as a 58.0% cardiothoracic ratio. Electrocardiogram revealed marked high-voltage QRS complexes, ST segment depressions in leads V_4–6_, inverted T-wave in leads _a_V_L_ and V_1–6_ and ST segment elevations in leads _a_V_R_ and V_1–2_ (Fig. 1A). Bone mineral density did not indicate excessive bone loss (a *Z*-score of −0.35 and a *T*-score of −0.35).

## Investigation

**Figure 2:**
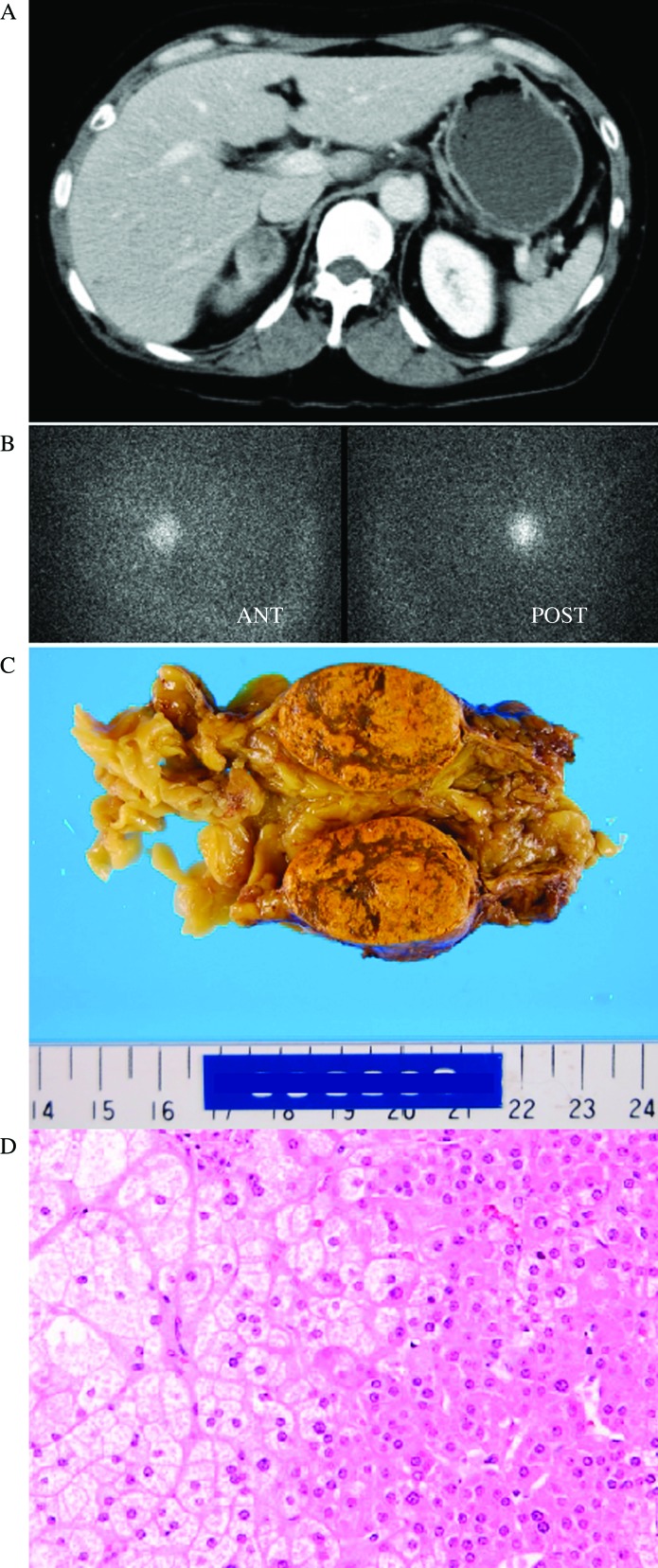
Abdominal computed tomography showing a 31×24 mm low-density mass within the right adrenal gland with mild enhancement post-contrast (A) that was also positive for [^131^I]adosterol imaging (B). Cut sections of the yellow adrenocortical adenoma (30×30×20 mm, 25 g nodule) with the atrophic right adrenal gland (C). Pathological evaluation of the right adrenal mass indicated diagnostic characteristics of a typical adrenocortical adenoma with Cushing's syndrome (D).

A history of the symptoms and physical features strongly suggested the presence of chronic glucocorticoid excess. The patient's serum potassium levels were normal (3.9 mEq/l) without administration of any medication such as angiotensin-converting enzyme inhibitor, angiotensin receptor type 1 blocker, aldosterone antagonist, calcium channel blocker and sodium-wasting diuretics. Other laboratory findings revealed a loss of the circadian rhythm of cortisol secretion (0900 h, 19.8 μg/dl; 1600 h, 20.9 μg/dl and 2300 h, 17.1 μg/dl) and elevated urinary free cortisol levels (112–118 μg/day; reference value, 11.2–80.3 μg/day) with no suppression after low (1 mg)- and high (8 mg)-dose dexamethasone tests (20.2 and 18.4 μg/dl respectively). ACTH levels were consistently suppressed (<2.0 pg/ml). Plasma renin activity (PRA; 0.5 ng/ml per h) and plasma aldosterone concentration (PAC; 40.4 pg/ml) were within the normal limits and the PAC-to-PRA ratio (80.8) was <200. Diabetes mellitus was ruled out according to the current Japan Diabetes Society's guideline using a 75 g oral glucose tolerance test. CT of the abdomen also revealed a 31×24 mm low-density mass within the right adrenal gland with a regular border and mild enhancement post-contrast (Fig. 2A). The right adrenal lesion was positive for [^131^I]adosterol imaging; however, the left adrenal gland was negative (Fig. 2B), suggesting that the right adrenal mass was responsible for ACTH-independent Cushing's syndrome in the patient.

Parasternal echocardiography revealed that the LV mass index (185 g/m^2^) and the relative wall thickness (0.75) were high and the interventricular septum was remarkably thick (20.1 mm) (Fig. 1B), indicating LV concentric hypertrophy concomitant to asymmetric septal thickness (interventricular septum thickness in diastole (IVSd)/posterior wall thickness in diastole=1.42). Mid-wall fractional shortening (FS) (13.7%) but not endocardial FS (43.2%) was low, suggesting impaired systolic performance with a disturbed inward movement of the thickened LV inner layer. The E/A ratio was low (0.63), and the E-wave deceleration time was prolonged (213 ms), both of which were also indicative of abnormal diastolic relaxation and filling velocities.

## Treatment

The patient underwent laparoscopic right adrenalectomy (Fig. 2C). Histology confirmed the diagnosis of a benign adrenocortical adenoma by the mixture of clear cells of the zona fasciculata type and compact cells of the zona reticularis type (Fig. 2D). After the surgery, the patient made an uneventful recovery, although glucocorticoid coverage was transiently required. The recovery of normal function of the hypothalamic–pituitary axis was assumed from plasma ACTH levels that exceeded the upper limit of the reference range (>63.3 pg/ml). At 18 months after adrenalectomy, no symptoms were observed and plasma cortisol levels were 11.8 μg/dl at 0900 h. Subsequently, a rapid ACTH stimulation test using tetracosactide (250 μg intravenously) was performed, which revealed a plasma cortisol level of 16.5 μg/dl at 30 min after the injection; therefore, adrenal replacement therapy was discontinued.

## Outcome and follow-up

**Figure 3:**
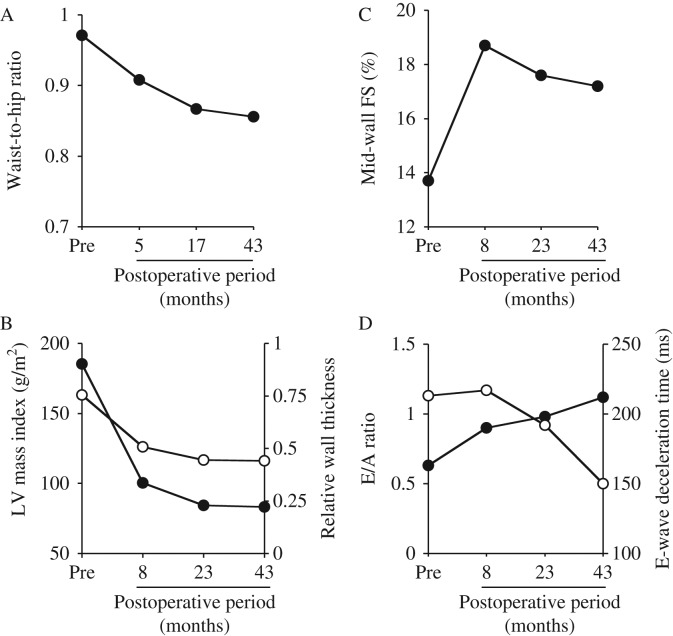
Serial measurements of waist-to-hip ratio and from echocardiography before and after adrenalectomy. The patient's waist-to-hip ratio was periodically assessed and was found to be gradually decreased at 5, 17 and 43 months after adrenalectomy (A). Follow-up examinations showed significant regression of the LV mass index (closed circles) and relative wall thickness (open circles) (B), improvement of mid-wall fractional shortening (C) and recovery of the E/A ratio (closed circles) and reduction of the E-wave deceleration time (open circles) (D) at 8, 23 and 43 months after the surgery.

The surgical treatment was effective on features of Cushing's syndrome, with a relief of the physical signs. Regular menstrual cycles (25 days/cycle) resumed 3 months after adrenalectomy. The patient had gradually lost 10.5% of the initial body weight (body weight, 45.8 kg; BMI, 20.4; waist circumference, 67.9 cm and waist-to-hip ratio, 0.87) up to 17 months after adrenalectomy and maintained the loss for >3 years (Fig. 3A), suggesting that hypercortisolism may have, at least partially, affected her body weight, concomitant with the relative central fat accumulation.

The abnormal electrocardiogram findings promptly disappeared at 8 months after adrenalectomy (Fig. 1C). The cardiothoracic ratio in the chest X-ray was decreased by 8 months after adrenalectomy and this was sustained for >3 years after treatment (58.0% at the preoperative period, 48.6% at 8 months, 46.5% at 18 months, 45.2% at 27 months and 45.1% at 39 months after adrenalectomy). Follow-up echocardiography for analysis of the postoperative cardiac course was performed at 8, 23 and 43 months after complete biochemical remission from hypercortisolism. The thickness of the posterior wall decreased, and the asymmetric septal hypertrophy almost disappeared within 8 months after the surgery (Figs 1D and 3B). Consistent with the changes in the LV structure, mid-wall FS, E/A ratio and E-wave deceleration time remarkably improved at the same point of time (Fig. 3C and D).

**Figure 4:**
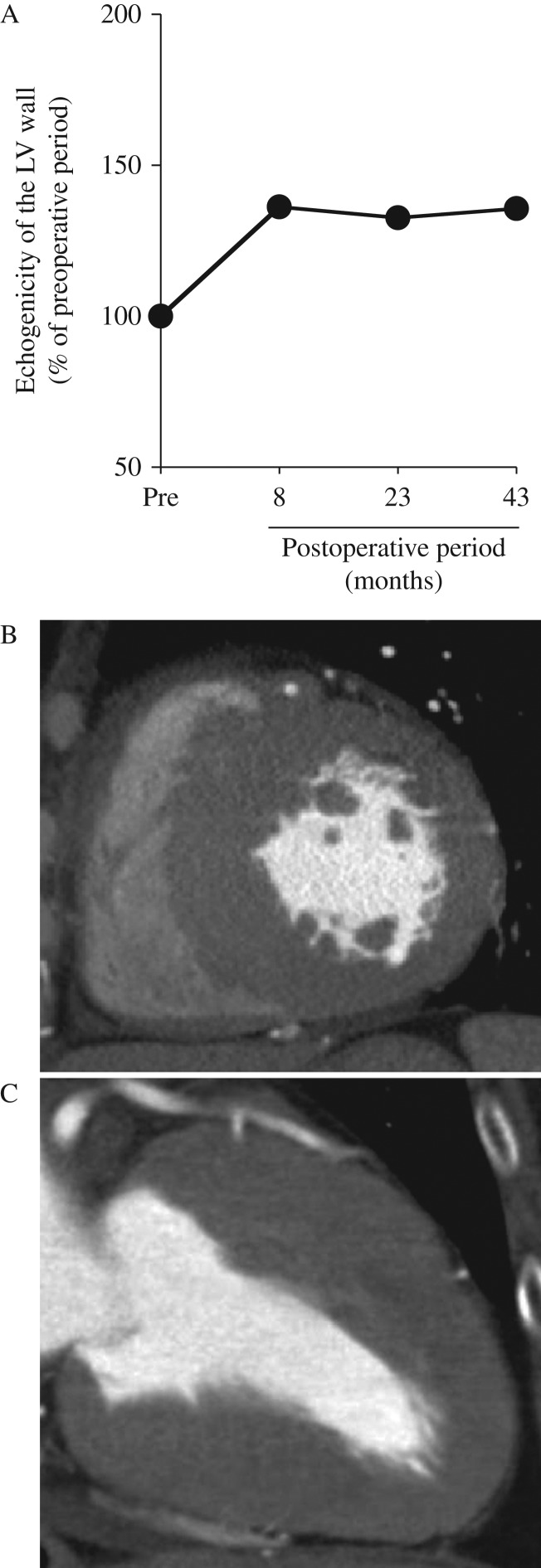
Change in ultrasound reflectivity and characteristics of CT signal density of the left ventricular wall. A 9×9 mm region of interest imaged by echocardiography was positioned in the mid-myocardium of the anteroseptal and posterior walls, and a 2×2 mm region of interest was positioned in the pericardium for reference. The ultrasound reflectivity of the regions was quantified using the NIH Image Software (Image J version 1.2; National Institutes of Health, Bethesda, MD, USA). The values in the graph (closed circles) represent the relative levels of echogenicity of the anteroseptal and posterior walls to those of the pericardium (A). Short-axis (B) and axial images (C) by contrast-enhanced cardiac multidetector-row CT showed myocardial hypoenhancement in the anteroseptal wall before the treatment.

To investigate whether surgical remission of hypercortisolism affects myocardial characteristics, ultrasound reflectivity of the LV anteroseptal and posterior walls was quantified by densitometry. Serial echocardiography revealed that echogenicity in the LV anteroseptal and posterior walls at 8 months after adrenalectomy was higher than that during the untreated active state by 36% and that the level of intensity was sustained for 43 months after the treatment (Fig. 4A). Contrast-enhanced multidetector-row CT revealed the presence of anteroseptal wall thickness with hypoenhancement of the myocardium, consistent with fatty infiltration (24–48 Hounsfield Unit) before the treatment (Fig. 4B and C).

## Discussion

This report describes a patient with Cushing's syndrome, who presented with a specific cardiac morphology characterised by disproportional hypertrophy of LV with an impaired systolic performance and relaxation, as shown by the mid-wall FS, E/A ratio and E-wave deceleration time. These findings are predictive of the occurrence of subsequent cardiovascular events and heart failure (7) (8). Mid-wall FS was found to be largely within the normal range at 8 months after adrenalectomy. In addition, the E/A ratio and E-wave deceleration time gradually improved during 43 months after the treatment, which may provide relief from adverse cardiovascular events in the future.

Approximately 80% of patients with Cushing's syndrome have high blood pressure and present with a more severe alteration of cardiac mass parameters compared with normotensive patients with Cushing's syndrome (1) (4). Before and during the admission, the blood pressure of the patient did not meet the Japanese Society of Hypertension's criteria for the diagnosis of hypertension without antihypertensive drugs, although the absence of nocturnal blood pressure dipping was not evaluated using 24 h ambulatory blood pressure values. It is estimated that 70% of patients with Cushing's syndrome have diabetes or glucose intolerance that also contributes to LV mass alterations (1) (4). A possible influence of glucose intolerance on cardiac structure and functions was excluded by the glucose tolerance test, suggesting a direct effect of cortisol on the cardiac morphology in this case.

In five previous case reports, severe congestive heart failure associated with Cushing's syndrome was improved by medical and surgical treatment for hypercortisolism (9) (10) (11) (12) (13). All the patients were required to undergo pre- and postoperative treatment for congestive heart failure with medications such as diuretics, αβ blockers, angiotensin-converting enzyme inhibitors, angiotensin receptor blockers, aldosterone antagonists and/or antidiabetic agents. These treatments, however, may have contributed to a reversible cardiac morphology and function independent of correcting hypercortisolism. In view of this, the present patient was treated with adrenalectomy alone without any medication except adrenal hormones for postoperative adrenal insufficiency; this may therefore convincingly lend credence to the relationship between hypercortisolism and progression of unique cardiac remodelling.

In contrast to the present patient's manifestation of severe concentric LV hypertrophy with asymmetric septal thickness, the patients in the above five cases presented dilated cardiomyopathy without apparent septal hypertrophy (IVSd, 8.6 and 13 mm in the two reports (10) (13)). Among them, two cases involved excessive cortisol levels; the urinary free cortisol level of one patient was almost equal to that of the present patient (112 μg/day) (12), whereas that of another patient was much higher (249 μg/day) (13). Although the exact reason(s) for the difference in cardiac morphology between the present case and previous cases remain(s) unclear, the degree of cortisol excess is unlikely to be responsible for the asymmetric LV hypertrophy, as reported previously (6).

Assessment using ultrasonic integrated backscatter has demonstrated that myocardial fibrosis is significantly increased in active, untreated patients with Cushing's syndrome and is related to LV systolic and diastolic dysfunctions (5). The pericardium contains the highest content of fibrosis and shows the highest ultrasound reflectivity within the heart. Therefore, a higher level of echogenicity in the myocardium represents the progression of myocardial fibrosis. The multiple spatial sampling by echocardiography revealed that surgical remission of hypercortisolism for 8 months resulted in a prompt 36% increase in ultrasound reflectivity in the mid-myocardium of the anteroseptal and posterior wall compared with that during the untreated active state and that the level of echogenicity was sustained for 43 months after adrenalectomy, suggesting that myocardial fibrosis may play a minor role in the alteration of the cardiac morphology in the patient.

Cushing's syndrome is characterised by abnormal fat deposition in subcutaneous sites on the face, neck, abdomen and skeletal muscle (1). Muscle fibre wasting and fat replacement are implicated in myopathy with proximal weakness that is frequently observed in Cushing's syndrome (14). Excess lipid accumulation in the myocardium has been observed in obese humans with initial cardiomyocyte hyperplasia preceding increased myocardial fibrosis, followed by myofilament loss (15). Therefore, failure of liporegulation and ectopic fat deposition in the myocardium may be related to LV remodelling in this case. Indeed, the present case revealed that relative wall thickness was regressed after adrenalectomy, paralleling the decrease in central fat accumulation represented by the patient's weight and waist-to-hip ratio. Echocardiography may be limited to the differentiation of tissue characteristics; however, the myocardial density of the contrast-enhanced multidetector-row CT image before adrenalectomy suggested fatty infiltration of the myocardium in the thickened LV wall.

Sugihara *et al*. (16) also reported three cases of Cushing's syndrome in which patients exhibited similar LV structure and functions. Right endocardial biopsies at the preoperative period were performed in two cases. Mild hypertrophy of myocytes was observed in one patient in whom cardiac functions were normalised by 8 months after surgical remission of hypercortisolism. The other patient showed mild degeneration of myocytes with an increase in interstitial fibrosis and failed to fully recover cardiac function even >3 years after adrenalectomy. It has been reported that surgical remission fails to significantly reverse cardiac mass alteration or recover cardiac function indices in the majority of patients with Cushing's syndrome (4). However, Pereira *et al*. (17) demonstrated that patients with Cushing's syndrome have abnormalities of LV structure and diastolic function, which are reversible upon normalisation of excessive cortisol levels. This discrepancy in outcomes may be attributed to the difference in the progress of myocardial degeneration. Impaired cardiac function in patients with Cushing's syndrome is reversible by biological remission when the myocardium remains in the early phase of cardiomyocyte hyperplasia with excessive lipid accumulation, although it may be irreversible in the late phase of advancing myocardial fibrosis. Early diagnosis and treatment of Cushing's syndrome for preventing advanced myocardial fibrosis are therefore pivotal for recovering cardiac functions and obtaining a favourable outcome.

## Patient consent

Written informed consent was obtained from the patient for publication of this case report.

## Author contribution statement

H Iwasaki was the patient's physician, was responsible for case description and literature review and wrote the manuscript.
